# *HFE* p.S65C (rs1800730) in Europe: A Geographic Study

**DOI:** 10.3390/genes17080951

**Published:** 2026-08-14

**Authors:** James C. Barton, J. Clayborn Barton, Ronald T. Acton

**Affiliations:** 1 Southern Iron Disorders Center, Birmingham, AL 35209, USA; jbarton205@gmail.com (J.C.B.); acton@mail.ad.uab.edu (R.T.A.); 2 Department of Medicine, The University of Alabama at Birmingham, Birmingham, AL 35294, USA; 3 Department of Microbiology, The University of Alabama at Birmingham, Birmingham, AL 35294, USA

**Keywords:** fitness, hemochromatosis, *HFE* p.S65C heterozygote, iron overload, latitude, longitude

## Abstract

Background: *HFE* (homeostatic iron regulator) p.S65C (rs1800730) is associated with European ancestry, although there is no comprehensive geographic study of p.S65C in Europe. Methods: We tabulated published p.S65C genotypes in European population/control cohorts, depicted allele frequency ranges in a map, and computed correlations of p.S65C allele frequencies with latitudes and longitudes of cohort recruitment sites. Results: We identified 44 cohorts from 24 of the 44 countries of Europe. These 24 countries comprise ~62.9% of Europe’s population. There were 233,528 subjects, including 38 *HFE* p.S65C homozygotes (~1 in 6145 subjects) and 4347 p.S65C heterozygotes (~1 in 54 subjects). Genotypes in 42 cohorts (95.5%) were consistent with Hardy–Weinberg equilibrium. The median allele frequency was 0.0129 (range 0–0.0350). p.S65C was not reported in Bulgaria, although the allele frequency in non-Bulgarian cohorts did not differ significantly (0/200 vs. 4423/462,432, respectively; *p* = 0.2728). Allele frequency ranges (means) were 0–0.0100 (0.0087), 0.0101–0.0200 (0.0146), and ≥0.0201 (0.0253) in 7, 14, and 3 respective aggregate country cohorts (*p* < 0.0001, all mean comparisons). The seven countries with low-range frequencies extend from north to south (Estonia to Italy). The three countries with high-range frequencies are east of ~14.1° E (western-most border of Poland). Correlations of allele frequencies with latitudes and longitudes in 44 cohorts were not significant (*p* = 0.1745 and 0.6031, respectively). Conclusions: We conclude that the median *HFE* p.S65C allele frequency in Europe is 0.0129, allele frequencies differ between some countries, and there is no linear north-to-south or west-to-east allele frequency gradient.

## 1. Introduction

*HFE*, the homeostatic iron regulator (chromosome 6p22.2), encodes HFE, a non-classical class I major histocompatibility complex (MHC)-related transmembrane glycoprotein [1]. The peptide-binding groove of the HFE α1 domain binds transferrin receptor-1 (TfR1) [2,3]. TfR1 binding competes with the binding of iron-loaded transferrin at the adjacent HFE α2 peptide-binding groove [4], thus modulating iron uptake in hepatocytes [5]. HFE is also an upstream modulator of the hepatic synthesis of hepcidin, the master regulator of iron homeostasis [6].

*HFE*, originally designated as *HLA-H*, was discovered in 1996 among 178 US patients of European ancestry with hemochromatosis phenotypes [7]. Among them, 83.1% were homozygous for the mutation p.C282Y (exon 4, c.845G>A; rs1800562), 4.5% were compound heterozygotes for p.C282Y/p.H63D (exon 2, c.187C>G; rs1799945), and 0.6% were p.H63D homozygotes [7]. No mutation was identified in the remaining 11.8% [7]. Thus, *HFE*-related hemochromatosis is genetically heterogeneous.

A 1999 study of French hemochromatosis kinships discovered twin brothers without hemochromatosis phenotypes who were *HFE* p.C282Y/p.S65C (exon 2, c.193A>T; rs1800730) compound heterozygotes [8]. This suggested at first that p.S65C did not contribute to the development of hemochromatosis phenotypes. A subsequent study of 711 unrelated French hemochromatosis patients revealed that the prevalence of p.S65C was significantly increased in patients with at least one chromosome 6p without either p.C282Y or p.H63D, and that iron overload in such patients was typically mild [9]. p.S65C alters the HFE α1 peptide-binding groove by encoding a larger cysteine instead of a smaller serine moiety [2]. Serine-65 is conserved in HFE orthologs in other mammalian species [10].

Most patients with hemochromatosis iron phenotypes reported in early studies were Europeans [11,12]. *HFE* p.C282Y and p.H63D genotyping of control subjects in global population cohorts confirmed that p.C282Y occurs almost exclusively in persons of European descent, whereas p.H63D is cosmopolitan [13]. Subsequent studies revealed that p.S65C, like p.C282Y, is also associated with European ancestry [9,14]. p.S65C allele frequencies in four European cohorts otherwise undefined were 0.0099–0.0221 (622,674 subjects) [15]. p.S65C is rare or absent in Africans, Asians, and Indigenous Americans [15,16].

*HFE* p.C282Y allele frequencies occur in significant linear gradients from north to south across Europe [17,18,19], although we found no comprehensive study of the geographic distribution of p.S65C allele frequencies in Europe.

The goals of this study were (1) to tabulate published *HFE* p.S65C genotypes and allele frequencies in European population/control cohorts, (2) to depict p.S65C allele frequency ranges in a map of Europe, and (3) to compute linear correlations of p.S65C allele frequencies with the latitudes and longitudes of cohort recruitment sites. We discuss the differences and geography of p.S65C allele frequencies in European countries, the origin and age of p.S65C, the iron phenotypes associated with p.S65C genotypes, and the possibility that p.S65C heterozygosity conferred a fitness advantage.

## 2. Methods

### 2.1. Europe and European Countries

We defined Europe as the geographic region with the following borders: the Arctic Ocean in the north; the Ural Mountains, the Ural River, the Caspian Sea, the Caucasus Mountains, and the Black Sea in the east; the Mediterranean Sea in the south; and the Atlantic Ocean in the west [20]. We defined European countries as the 44 sovereign European nations recognized by the United Nations [20]. We included the area of Kosovo as part of Serbia [20].

### 2.2. Definition of Evaluable Population/Control Cohorts

We performed computerized and manual searches using the terms *HFE* mutation, *HFE* p.S65C, rs1800730, hemochromatosis, and country names. We defined reports of evaluable population/control cohorts [19] as those that included the following: 50 or more subjects [21], geographic recruitment site(s), and numbers of p.S65C genotypes. We included all evaluable cohorts.

### 2.3. Evaluable Population/Control Cohorts

We identified cohorts from Albania, Bulgaria, Croatia, Czechia, Denmark, Estonia, Finland, France, Germany, Greece, the Republic of Ireland, Italy, Lithuania, the Republic of North Macedonia, Montenegro, Poland, Portugal, Romania, Serbia, Slovakia, Slovenia, Spain, Sweden, and the United Kingdom (England only). We tabulated the latitudes and longitudes of the respective cohort recruitment sites [22].

### 2.4. Population/Control Cohorts Not Identified

We did not identify cohorts from Andorra, Austria, Belarus, Belgium, Bosnia and Herzegovina, Holy See (Vatican City), Hungary, Iceland, Latvia, Liechtenstein, Luxembourg, Malta, Monaco, Netherlands, Norway, the Republic of Moldova, the Russian Federation (European region), San Marino, Switzerland, Ukraine, and three constituent countries of the United Kingdom (Northern Ireland, Scotland, and Wales).

### 2.5. Population/Control Cohorts Excluded

We excluded subjects recruited in dependencies and autonomous or island territories of the 44 European sovereign nations, cohorts of non-European subjects who resided in Europe, and subjects selected because they had high-iron or hemochromatosis phenotypes.

### 2.6. Genotype Definitions

We defined *HFE* p.S65C homozygotes as subjects with the genotype p.S65C/p.S65C. We defined p.S65C heterozygotes as subjects with simple p.S65C heterozygosity or p.S65C/p.C282Y or p.S65C/p.H63D compound heterozygosity.

### 2.7. rs1800730 in Ancient Human DNA

We searched the SNP database of the Allen Ancient DNA Resource [23,24] to identify individuals with rs1800730. We also reviewed selected articles that report DNA analyses of ancient Europeans.

### 2.8. Estimated Age of rs1800730

We searched chromosome 6 SNPs in the database Atlas of Variant Age [25,26] for a rs1800730 entry.

### 2.9. Statistics

All data we analyzed are displayed herein. These data represent 44 European country/region cohorts and 24 countries of Europe. We tabulated *HFE* p.S65C genotypes, total p.S65C alleles, and total alleles in each cohort based on our interpretations of published reports. In a report of p.S65C alleles in the combined region of Serbia/Montenegro [27], we attributed one-half of the cohort and the same allele frequency to each country. We attributed Norwich, England cohort data [28] to the United Kingdom as a country/region cohort and to England only for mapping.

We used exact tests [29] with mid-p adjustments [30] to determine whether the numbers of subjects with *HFE* p.S65C homozygosity, p.S65C heterozygosity, and no p.S65C in each cohort were consistent with Hardy–Weinberg equilibrium (HWE).

We computed the *HFE* p.S65C allele frequency of each cohort, the median (range) and aggregate allele frequency of all 44 cohorts [95% confidence interval (CI)], and the aggregate country allele frequencies (24 countries). We compared allele proportions using Fisher’s exact test (two-tailed) or the chi-square test (two-tailed), as appropriate.

We grouped the 24 aggregate country *HFE* p.S65C allele frequencies in the following low, intermediate, and high ranges: 0–0.0100, 0.0101–0.0200, and ≥0.0201, respectively. We depicted the same allele frequency ranges in the present map of Europe.

The 44 *HFE* p.S65C country/region allele frequencies and longitudes in decimal degrees (four decimal places) [22] were normally distributed (Kolmogorov–Smirnov test; *p* = 0.2988 and *p* = 0.3497, respectively), whereas the corresponding latitudes in decimal degrees (four decimal places) [22] were not normally distributed (*p* < 0.0001). Thus, we computed a Pearson’s correlation (r; two-tailed) of p.S65C allele frequencies with longitudes and Spearman’s rank correlation (ρ; two-tailed) of p.S65C allele frequencies with latitudes. We defined significant correlations as linear allele frequency gradients.

We used Excel^®^ 2000 (Microsoft Corp., Redmond, WA, USA), GraphPad Prism 8^®^ (2018; GraphPad Software, San Diego, CA, USA), and MapChart^®^ 2026 [31]. We defined values of *p* < 0.05 to be significant.

## 3. Results

### 3.1. Population/Control Cohorts

We identified 44 cohort reports (233,528 subjects) from 24 of the 44 countries of Europe. These 24 countries comprise ~62.9% of Europe’s population [32]. There were 38 *HFE* p.S65C homozygotes (~1 in 6145 subjects) and 4347 p.S65C heterozygotes (~1 in 54 subjects) (Table A1). Of the 4385 subjects with p.S65C, 4347 (99.1%) were p.S65C heterozygotes. p.S65C genotypes in 42 cohorts (95.6%) were consistent with HWE, whereas p.S65C genotypes in the Estonian [33] and Greek [34] cohorts were not consistent with HWE (Table A1).

### 3.2. HFE p.S65C Allele Frequencies in Europe

The median *HFE* p.S65C allele frequency in 44 cohorts was 0.0129 (range 0–0.0350). The aggregate allele frequency in 233,528 subjects was 0.0095 [95% CI: 0.0092, 0.0098] (Table A1). p.S65C was detected in each of the 24 country/region cohorts except Bulgaria/Sofia (0.0000 [0, 0.0188]) (Table A1). The allele frequency of the Bulgarian cohort and the aggregate allele frequency of the non-Bulgarian cohorts did not differ significantly (0/200 vs. 4423/462,432, respectively; *p* = 0.2728).

Approximately 92.6% of the present subjects were Estonians. The aggregate *HFE* p.S65C allele frequency of the non-Estonian cohorts (0.0149 [0.0138, 0.0161]) was higher than that of the Estonian cohort (0.0089 [0.0086, 0.0092]) (640/43,068 vs. 3783/423,988, respectively; *p* < 0.0001).

### 3.3. HFE p.S65C Allele Frequencies in 24 European Countries

Aggregate *HFE* p.S65C allele frequencies of 24 country cohorts differed more than 15.1-fold (Table 1). Aggregate allele frequency ranges were low (0–0.0100) in 7 country cohorts, intermediate (0.0101–0.0200) in 14 country cohorts, and high (≥0.0201) in 3 country cohorts (Table 1). p.S65C allele frequencies were lowest in Bulgarian and Danish cohorts and highest in Macedonian and Polish cohorts. The aggregate allele frequency was ≥0.0101 in 17 of 24 countries (70.8%).

The aggregate *HFE* p.S65C allele frequencies in the low-, intermediate-, and high-range cohorts were 0.0087 (3871/444,304), 0.0146 (311/21,368), and 0.0253 (24/950), respectively. There were significant differences between these three allele frequencies (3 × 2 chi-square = 106.1; *p* < 0.0001). Subsequent 2 × 2 chi-square analyses for each of the three comparisons revealed *p* < 0.0001.

### 3.4. Map of HFE p.S65C Allele Frequencies in Europe

**Figure 1:**
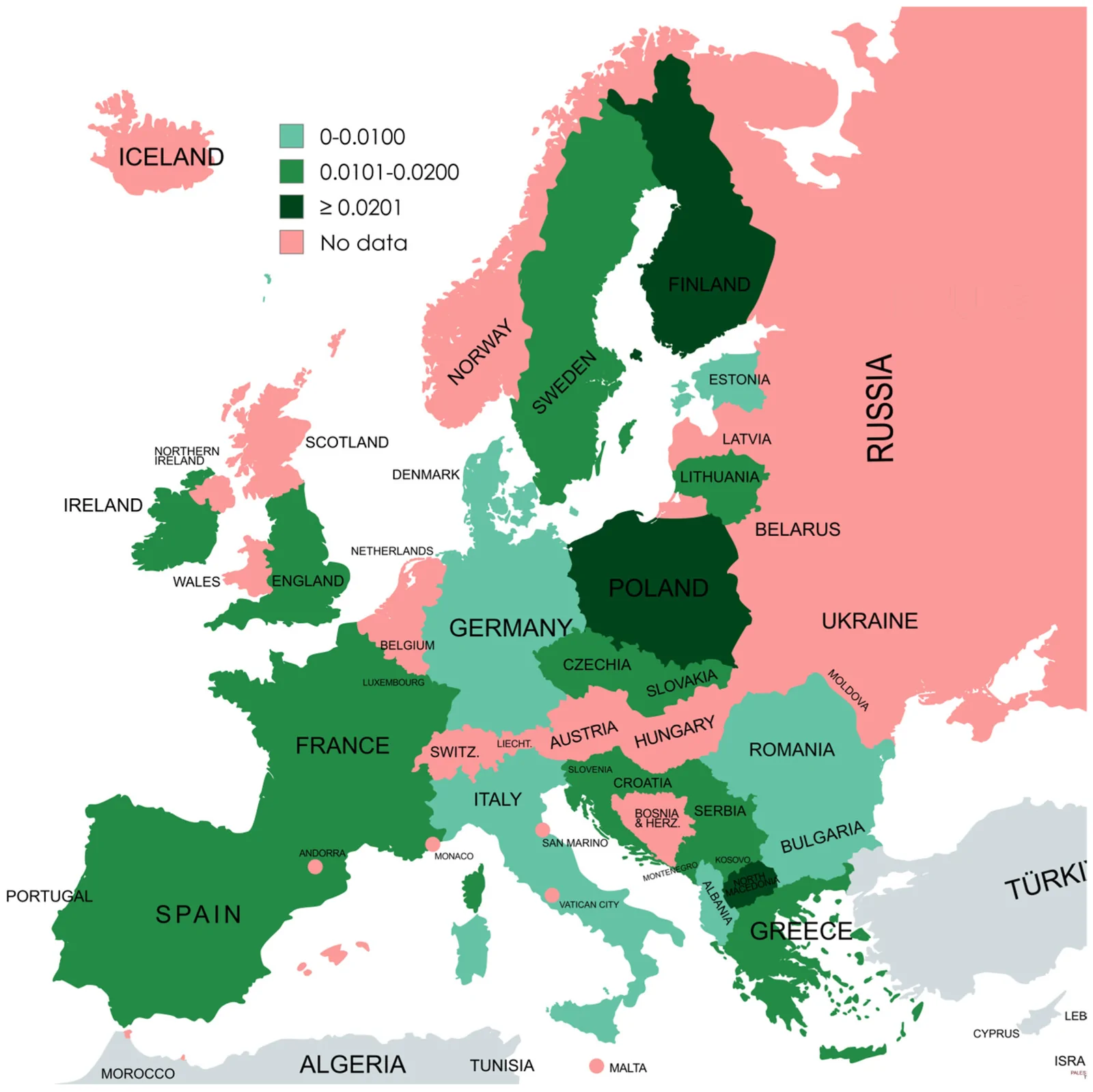
Aggregate *HFE* p.S65C allele frequencies in 24 countries of Europe.

*HFE* p.S65C allele frequencies of low, intermediate, and high range are scattered (Figure 1). The seven countries with aggregate frequencies in the low range (0–0.0100) extend from north to south (Estonia to Italy) (Table 1; Figure 1). The three countries with aggregate p.S65C allele frequencies in the high range (≥0.0201) are east of ~14.1° E (western-most border of Poland) (Table 1; Figure 1).

### 3.5. HFE p.S65C Allele Frequencies, Latitude, and Longitude

**Figure 2:**
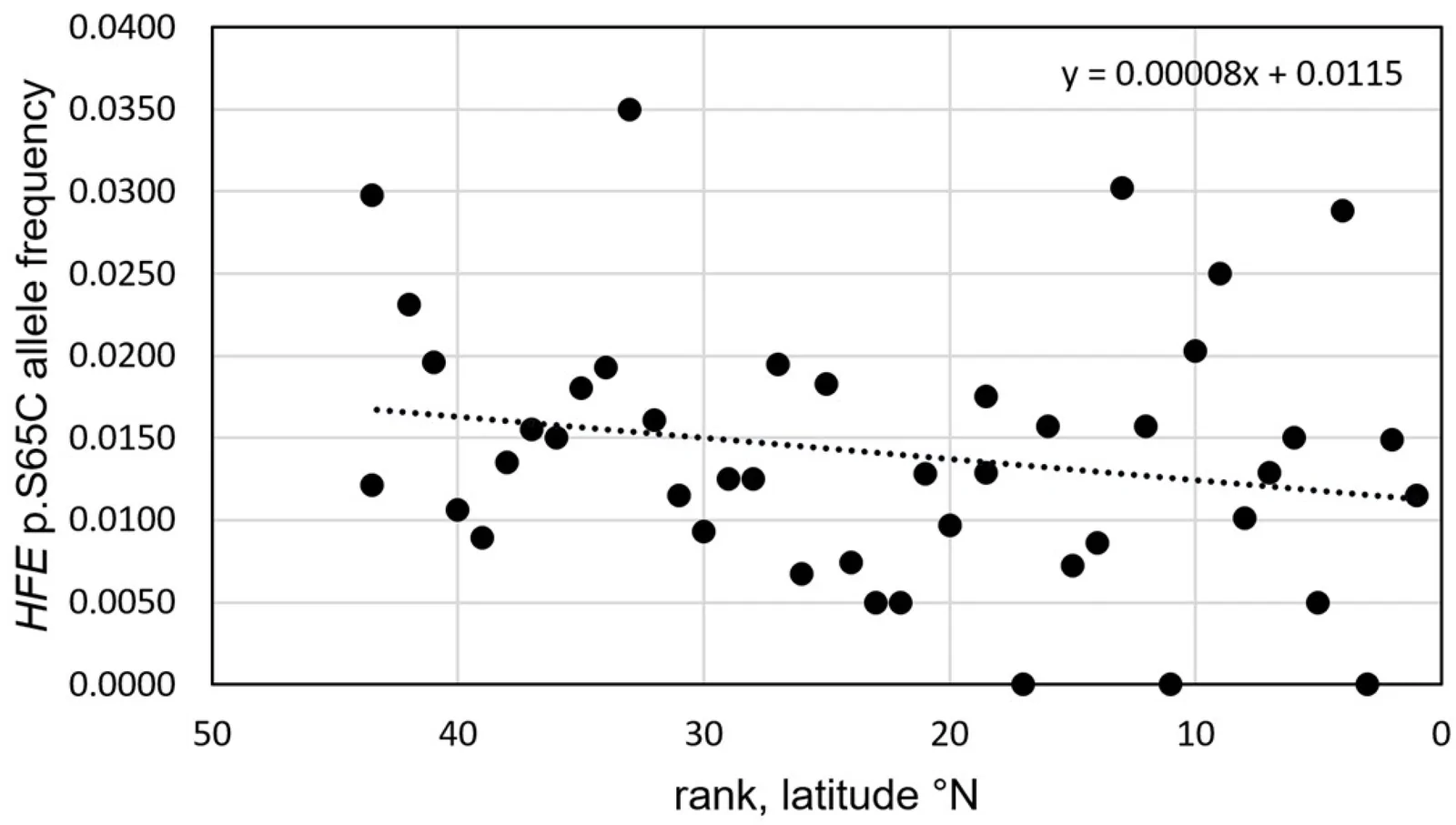
Spearman’s rank correlation of *HFE* p.S65C allele frequencies with latitudes in Europe (ρ_44_ = 0.2086; *p* = 0.1745 (two-tailed)).

**Figure 3:**
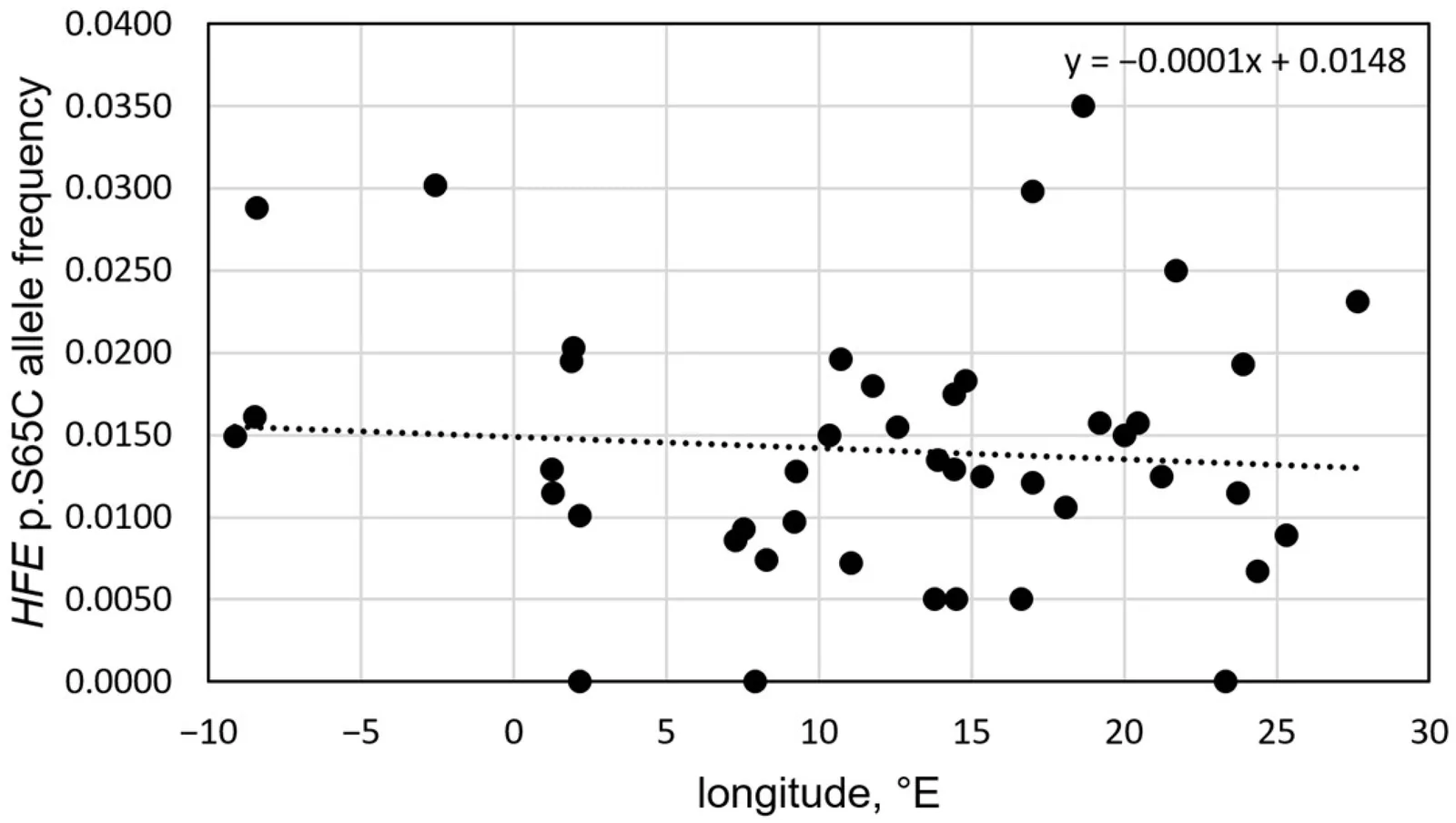
Pearson’s correlation of *HFE* p.S65C allele frequencies with longitudes in Europe (r_44_ = −0.0806; *p* = 0.6031 (two-tailed)).

Cohorts were recruited in this region: latitude 68.8776–37.9756° N; longitude −9.1366–27.6395° E (Table A2). The 24 countries we studied represent ~41.2% of Europe’s land area [35]. Spearman’s rank correlation of *HFE* p.S65C allele frequencies with latitudes in the 44 cohorts was not significant (*p* = 0.1745) (Figure 2). Pearson’s correlation of p.S65C allele frequencies with longitudes was not significant (*p* = 0.6031) (Figure 3).

The exclusion of the Estonian cohort did not change the correlations of *HFE* p.S65C allele frequencies with latitudes and longitudes greatly (ρ_43_ = 0.2380; *p* = 0.1243 and r_43_ = −0.0603; *p* = 0.7011, respectively).

### 3.6. rs1800730 in Ancient Human DNA

We did not find an entry for rs1800730 in the Allen Ancient DNA Resource of ~1.2 M SNPs [23,24]. Shotgun sequencing of the DNA from four ancient persons buried in Ireland identified the wild-type allele (*HFE* p.S65 (c.193A)) [36].

Factors that could influence the ability to detect rs1800730 in ancient DNA, if it were present, include the geographic site from which the DNA was recovered, DNA degradation and fragmentation [37], contamination with exogenous DNA [38], geographic and environmental factors that affect DNA stability [39], and the method(s) used to identify rs1800730, if any.

### 3.7. Estimated Age of rs1800730

We did not find an entry for rs1800730 in the 26,572 chromosome 6 SNPs in the database Atlas of Variant Age [25,26].

## 4. Discussion

The median *HFE* p.S65C allele frequency in 44 European cohorts in this study was 0.0129. This is consistent with allele frequencies in four European cohorts otherwise undefined (allele frequency range of 0.0099–0.0221) (622,674 subjects) [15]. Thus, p.S65C is classified as a rare allele (minor allele frequency <1.0% [40]) in some European countries and as a low-frequency allele (minor allele frequency 1.0–5.0% [40]) in other European countries.

Factors that could contribute to *HFE* p.S65C allele frequency differences across Europe include gene flow, genetic drift, different selection criteria for different population/control cohorts [41], small cohorts [42], and cultural factors [43]. The most likely causes of significant deviation from HWE proportions in the two present cohorts are sampling bias [44] and genotyping errors [45].

The present map depicts a mosaic of *HFE* p.S65C allele frequency ranges in Europe. There was no linear gradient of frequencies from either north to south or west to east. The distribution of p.S65C throughout Europe could be explained by a selective advantage of p.S65C, founder effects and bottlenecks, and genetic drift [46].

*HFE* p.S65C is confined predominantly to Europe and derivative countries [1] and thus probably arose in an ancient European, although we found no documentation of rs1800730 in ancient DNA. Archaeological evidence indicates that *Homo sapiens* was present in Europe 56,000–43,000 years ago [47,48,49,50]. This interval suggests the approximate maximum age of p.S65C, although we found no published estimate of the age of rs1800730.

The ancestral chromosome 6p haplotype on which *HFE* p.S65C arose is unknown. In Madrid, Spain, p.S65C occurs in association with the human leukocyte antigen (HLA) locus -A*26 [51]. In Terceira Island, Azores, Portugal, p.S65C occurs on the HLA haplotype -A*29, B*44 [52]. In Alabama, USA, p.S65C occurs in association with HLA-A*32 [53]. Multiple chromosome 6p recombination events that separated p.S65C from its ancestral HLA haplotype could explain these observations. It is also plausible that p.S65C arose in multiple *HFE* mutational events [54], each on a different HLA haplotype.

The mean iron saturation of serum transferrin in subjects with p.S65C heterozygosity without another *HFE* mutation and that of subjects with *HFE* wt/wt (absence of *HFE* p.C282Y, p.H63D, or p.S65C) did not differ significantly [14,55,56,57,58]. The prevalence of *HFE* p.S65C genotypes in Swedish patients with elevated levels of both serum transferrin saturation and serum ferritin and in control subjects did not differ significantly [14]. In US non-Hispanic white participants in a population-based screening program, p.S65C allele frequencies in participants with elevated levels of both serum transferrin saturation and serum ferritin and in control participants did not differ significantly [16].

Mild or moderate iron overload demonstrated by assessments of liver iron content or phlebotomy has been reported in some *HFE* p.S65C heterozygotes, p.S65C/p.C282Y and p.S65C/p.H63D compound heterozygotes, and p.S65C homozygotes [14,53,59,60]. Thus, iron absorption is increased in some persons with p.S65C genotypes.

Screening for iron overload and hemochromatosis in European and derivative populations using either iron phenotyping or *HFE* mutation analysis, including that for p.S65C, is not recommended [61,62,63]. Regardless of genotype, the penetrance of iron overload in persons with *HFE*-related hemochromatosis is low; results may cause anxiety and misunderstanding, and diagnostic errors may occur, all of which contribute to an ineffective expenditure of diagnostic resources [61,62,63].

Preferred diagnostic testing for hemochromatosis in individuals with high-iron phenotypes is iron phenotyping (transferrin saturation and serum ferritin) followed by *HFE* mutation analysis. Diagnostic testing for *HFE* mutations performed by major clinical diagnostic laboratories in the US [64,65] and another in Germany [66] includes p.C282Y, p.H63D, and p.S65C, whereas a major laboratory in Australia does not test for p.S65C [67]. Although the demonstration of p.S65C may fortify the diagnosis of *HFE*-related hemochromatosis in patients with high-iron phenotypes without p.C282Y homozygosity, p.H63D homozygosity, or p.C282Y/p.H63D compound heterozygosity [68], the decision to perform therapeutic phlebotomy is based on iron phenotyping alone [62,63]. The FinnGen genome-wide association study for hemochromatosis in 420,543 individuals demonstrated that “the S65C variant protects against severe [iron overload] disease (incidence ratio 0.328, 95% CI 0.192–0.562)” [69].

Expression of *HFE* p.S65C cDNA in transfected HEK 293 cells did not alter the affinity of HFE for TfR1 significantly or impair other HFE functions in comparison with control cells [70]. Alternative splicing of HFE results in exon 2 skipping in many tissues, especially the duodenum [71], the primary site of iron absorption. These observations could explain in part the very low penetrance of p.S65C to cause iron overload [14,33,69].

Was the relative fitness of ancient *HFE* p.S65C heterozygotes increased? The mildly elevated serum ferritin levels of some European control subjects with p.S65C heterozygosity [14] hint that p.S65C could decrease risks for iron deficiency, but we identified no study that compared the p.S65C allele frequencies in European adults with and without iron deficiency. It has been postulated that *HFE* mutations enhance physical performance via iron-related pathways [72], but the p.S65C allele frequency in French athletes who won international competitions and that in healthy control subjects did not differ significantly (7/340 vs. 4/438, respectively; *p* = 0.2256) [73].

Immune response genes linked to *HFE*, including the more than 200 HLA genes [74], may have increased the relative fitness of ancient [75] and modern [76] subjects, although the extended HLA/MHC haplotype(s) linked to p.S65C are unreported. In a large retrospective study, p.S65C heterozygotes had increased rates of skin pigmentation disorder reports, especially those of hyperpigmentation [33], although the pertinence of this observation to ancient Europeans and its present clinical significance, if any, are not reported. The p.S65C allele proportions in Sardinians aged 60 years and aged ≥100 years did not differ significantly (1/120 vs. 4/114, respectively; *p* = 0.2032) [77], suggesting that p.S65C does not increase longevity.

Uncertainties of this study include the possibilities that we overlooked published reports of evaluable European cohorts and that there is a non-linear association of *HFE* p.S65C allele frequencies with latitude or longitude in Europe. Exclusion of the large Estonian cohort [33] increased the present aggregate p.S65C allele frequency, whereas excluding the Estonian cohort made little change in the non-significant correlations of allele frequencies with latitudes and longitudes.

A limitation of this study is that we identified no published *HFE* p.S65C population/control cohort for 20 countries in Europe, although we estimated that those countries would represent only ~37.1% of Europe’s population [32]. If those cohort data were available, they are unlikely to change the depiction of p.S65C allele frequencies greatly in a map of European countries or the non-significance of the correlations of p.S65C allele frequencies with latitude and longitude.

Assessing all factors that could contribute to *HFE* p.S65C allele frequency heterogeneity in Europe, investigating reasons for failure of two of the present cohorts to meet HWE criteria, analyzing p.S65C allele frequency data from European dependencies and autonomous and island territories, and comparing the prevalences of high-iron phenotypes attributed to p.S65C in different countries/regions of Europe were beyond the scope of this study.

## 5. Conclusions

We conclude that the median *HFE* p.S65C allele frequency in Europe is 0.0129, that allele frequencies differ between some countries, and that there is no linear north-to-south or west-to-east allele frequency gradient.

## Figures and Tables

**Table 1:** *HFE* p.S65C allele frequency ranges in 24 countries of Europe.

**Country**	**Low Frequency (0–0.1000)**
Bulgaria	0.0000
Denmark	0.0023
Romania	0.0067
Estonia	0.0089
Italy	0.0089
Germany	0.0093
Albania	0.0100
*A* *ggregate* *allele frequency [95% CI]*	*0.0087 [95% CI: 0.0084, 0.0090]*
**Country**	**Intermediate Frequency** (**0.0101–0.0200**)
Greece	0.0115
United Kingdom/England	0.0115
Czechia	0.0125
Slovakia	0.0125
Spain	0.0128
Croatia	0.0145
Montenegro	0.0157
Serbia	0.0157
Republic of Ireland	0.0161
Slovenia	0.0165
Sweden	0.0166
France	0.0182
Lithuania	0.0193
Portugal	0.0196
*Aggregate allele frequency [95% CI]*	*0.0146 [95% CI: 0.0131, 0.0163]*
**Country**	**High Frequency** (**≥0.0201**)
Finland	0.0218
Republic of North Macedonia	0.0250
Poland	0.0350
*Aggregate allele frequency [95% CI]*	*0.0253 [95% CI: 0.0171, 0.0374]*

CI, confidence interval.

## Data Availability

All data analyzed in this study are presented herein.

## Abbreviations

The following abbreviations are used in this manuscript:

CI	Confidence interval
HLA	Human leukocyte antigen
HWE	Hardy–Weinberg equilibrium
MHC	Major histocompatibility complex
TfR1	Transferrin receptor-1

## Appendix A

**Table A1:** *HFE* p.S65C in 44 European country/region cohorts.

Country/Region	Homozygotes, n	Heterozygotes, n	No p.S65C, n	HWE Value of *p*	Allele Frequency [95% CI]	Reference
Albania	0	1	49	0.5000	0.0100 [0.0018, 0.0545]	[80]
Bulgaria/Sofia	0	0	100	0.5000	0.0000 [0.0000, 0.0188]	[81]
Croatia/Rijeka	0	7	193	0.5260	0.0175 [0.0085, 0.0357]	[82]
Croatia/Rijeka	0	9	341	0.5254	0.0129 [0.0068, 0.0243]	[83]
Czechia	0	12	469	0.5335	0.0125 [0.0072, 0.0217]	[84]
Denmark	0	18	581	0.5608	0.0150 [0.0095, 0.0236]	[85]
Denmark/Copenhagen	0	13	407	0.5450	0.0155 [0.0091, 0.0263]	[86]
Denmark/Naestved	0	217	5803	0.1989	0.0180 [0.0158, 0.0205]	[58]
Estonia	32	3719	208,243	0.0006	0.0089 [0.0086, 0.0092]	[33]
Finland/north	0	8	165	0.5397	0.0231 [0.0117, 0.0449]	[87]
Finland/Oulu	0	4	98	0.5147	0.0196 [0.0076, 0.0493]	[88]
France	0	16	394	0.5693	0.0195 [0.0120, 0.0314]	[9]
France/Nice	0	1	57	0.5000	0.0086 [0.0015, 0.0472]	[89]
Germany/North Rhine-Westphalia	0	1	53	0.5000	0.0093 [0.0016, 0.0507]	[90]
Greece/Athens	5	18	1196	<0.0001	0.0115 [0.0080, 0.0166]	[34]
Republic of Ireland/Sligo	0	8	241	0.5277	0.0161 [0.0082, 0.0314]	[91]
Italy/Apulia	0	5	495	0.5050	0.0050 [0.0021, 0.0117]	[92]
Italy/Brianza	0	29	1103	0.5830	0.0128 [0.0089, 0.0183]	[93]
Italy/Domodossola	0	9	597	0.5147	0.0074 [0.0039, 0.0140]	[94]
Italy/Emilia-Romagna	0	3	204	0.5036	0.0072 [0.0024, 0.0210]	[95]
Italy/Friuli	0	1	99	0.5000	0.0050 [0.0009, 0.0278]	[95]
Italy/Milan	0	5	253	0.5097	0.0097 [0.0042, 0.0225]	[96]
Italy/Piedmont	0	0	104	0.5000	0.0000 [0.0000, 0.0181]	[95]
Lithuania/Kaunas	0	39	972	0.6559	0.0193 [0.0142, 0.0263]	[97]
Montenegro	0	5	154	0.5157	0.0157 [0.0067, 0.0362]	[27]
Republic of North Macedonia	0	5	95	0.5249	0.0250 [0.0204, 0.0769]	[80]
Poland/Gdansk	0	7	93	0.5514	0.0350 [0.0171, 0.0705]	[98]
Portugal/Coimbra	0	3	49	0.5146	0.0288 [0.0098, 0.0813]	[99]
Portugal/Lisbon	0	3	98	0.5075	0.0149 [0.0051, 0.0428]	[100]
Romania/Transylvania	0	3	222	0.5034	0.0067 [0.0023, 0.0195]	[101]
Serbia	0	5	154	0.5157	0.0157 [0.0067, 0.0362]	[27]
Slovakia/Prešov	0	9	350	0.5247	0.0125 [0.0066, 0.0236]	[102]
Slovenia	0	47	1235	0.6746	0.0183 [0.0138, 0.0243]	[103]
Slovenia/Ljublijana	0	2	198	0.5013	0.0050 [0.0014, 0.0180]	[82]
Spain/Barcelona	0	21	1022	0.5483	0.0101 [0.0066, 0.0154]	[104]
Spain/Basque Country	0	7	109	0.5445	0.0302 [0.0147, 0.0610]	[105]
Spain/Murcia	0	15	355	0.5674	0.0203 [0.0123, 0.0332]	[106]
Spain/Tarragona	0	21	791	0.5614	0.0129 [0.0084, 0.0197]	[57]
Spain/Toledo	0	0	150	0.5000	0.0000 [0.0000, 0.0126]	[107]
Sweden/north	0	5	201	0.5121	0.0121 [0.0052, 0.0280]	[87]
Sweden/north ^a^	1	7	143	0.0598	0.0298 [0.0158, 0.0557]	[87]
Sweden/south	0	8	288	0.5234	0.0135 [0.0069, 0.0264]	[87]
Sweden/Stockholm	0	8	242	0.5276	0.0160 [0.0081, 0.0313]	[14]
United Kingdom/England/Norwich ^b^	0	23	977	0.5589	0.0115 [0.0077, 0.0172]	[28]
*All 44 cohorts*	*38*	*4347*	*229,143*	*n.a.*	*0.0095 [95% CI: 0.0092, 0.0098]*	

CI, confidence interval; HWE, Hardy–Weinberg equilibrium; n.a., not applicable. ^a^ Includes Saamis. ^b^ We identified no *HFE* p.S65C population/control cohort from Northern Ireland, Scotland, or Wales.

**Table A2:** *HFE* p.S65C frequency, latitude, and longitude of 44 European countries/regions.

Country/Region	p.S65C Allele Frequency	Latitude °N	Longitude °E
Albania	0.0150	41.0000	20.0000
Bulgaria/Sofia	0.0000	42.6977	23.3217
Croatia/Rijeka	0.0175	45.3268	14.4422
Croatia/Rijeka	0.0129	45.3268	14.4422
Czechia	0.0125	49.7439	15.3381
Denmark	0.0150	55.6702	10.3333
Denmark/Copenhagen	0.0155	55.6867	12.5701
Denmark/Naestved	0.0180	55.2328	11.7674
Estonia	0.0089	58.7524	25.3319
Finland/north	0.0231	62.8937	27.6395
Finland/Oulu	0.0196	59.9148	10.7075
France	0.0195	46.6034	1.8883
France/Nice	0.0086	43.7009	7.2684
Germany/North Rhine-Westphalia	0.0093	51.4789	7.5544
Greece/Athens	0.0115	37.9756	23.7348
Ireland/Sligo	0.0161	54.2721	−8.4751
Italy/Apulia	0.0050	40.9843	16.6210
Italy/Brianza	0.0128	45.6395	9.2788
Italy/Domodossola	0.0074	46.1152	8.2920
Italy/Emilia-Romagna	0.0072	44.5257	11.0394
Italy/Friuli	0.0050	45.6529	13.7805
Italy/Milan	0.0097	45.4642	9.1896
Italy/Piedmont	0.0000	45.0607	7.9235
Lithuania/Kaunas	0.0193	54.8982	23.9045
Montenegro	0.0157	42.7756	19.2022
Republic of North Macedonia	0.0250	41.6171	21.7168
Poland/Gdansk	0.0350	54.3483	18.6540
Portugal/Coimbra	0.0288	40.2112	−8.4295
Portugal/Lisbon	0.0149	38.7078	−9.1366
Romania/Transylvania	0.0067	46.5972	24.3740
Serbia	0.0157	44.8161	20.4592
Slovakia/Prešov	0.0125	49.0000	21.2392
Slovenia	0.0183	46.1199	14.8153
Slovenia/Ljublijana	0.0050	46.0500	14.5069
Spain/Barcelona	0.0101	41.3826	2.1771
Spain/Basque Country	0.0302	42.9912	−2.5543
Spain/Murcia	0.0203	41.6993	1.9765
Spain/Tarragona	0.0129	41.1172	1.2546
Spain/Toledo	0.0000	39.8559	2.1771
Sweden/north	0.0121	63.8776	17.0018
Sweden/north ^a^	0.0298	63.8776	17.0018
Sweden/south	0.0135	55.8475	13.8866
Sweden/Stockholm	0.0106	59.3251	18.0711
United Kingdom/England/Norwich	0.0115	52.6286	1.2924

^a^ Saamis.
